# The potential significance of hepcidin evaluation in progressive supranuclear palsy

**DOI:** 10.1002/brb3.3552

**Published:** 2024-06-21

**Authors:** Piotr Alster, Dagmara Otto‐Ślusarczyk, Alicja Wiercińska‐Drapało, Marta Struga, Natalia Madetko‐Alster

**Affiliations:** ^1^ Department of Neurology Medical University of Warsaw Warsaw Poland; ^2^ Department of Biochemistry Medical University of Warsaw Warsaw Poland; ^3^ Department of Infectious and Tropical Diseases and Hepatology Medical University of Warsaw Warsaw Poland

**Keywords:** atypical parkinsonism, hepcidin, progressive supranuclear palsy, PSP

## Abstract

**Introduction:**

Hepcidin is a peptide associated with controlling the distribution of iron in tissues. Growing interest is linked with its impact on neurodegenerative diseases, as disruption of the iron regulation may be considered an initiatory element of pathological protein accumulation. The possible impact of hepcidin was not previously sufficiently explored in progressive supranuclear palsy (PSP).

**Methods:**

Twelve patients with PSP–Richardson's syndrome (PSP–RS), 12 with PSP‐Parkinsonism Predominant (PSP‐P), and 12 controls were examined using Unified Parkinson's Disease Rating Scale—III part (UPDRS‐III) in OFF stage and analyzed in the context of hepcidin levels in the serum.

**Results:**

The work revealed increased levels of hepcidin in PSP–RS when compared to PSP‐P and controls. Moreover, hepcidin was found to be negatively correlated with UPDRS‐III results in PSP–RS, whereas positively in PSP‐P.

**Conclusion:**

The work may suggest a possible impact of hepcidin in PSP, possibly differing depending on its subtype.

## Graphical abstract

The association between hepcidin levels and parkinsonian syndrome in Progressive Supranuclear Palsy subtypes.

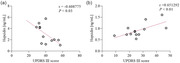

## 1 INTRODUCTION

Progressive supranuclear palsy (PSP) is the most common atypical parkinsonism. The most contemporary criteria of diagnosis, released in 2017, indicated numerous subtypes of the disease, which are classified according to the presence of main groups of symptoms, among which could be mentioned—akinesia, oculomotor dysfunction, cognitive/language deficits, and postural deficiencies (Höglinger et al., 2017). Though the criteria indicate the combination of symptoms associated with certain subtype, the examination of the diseases is affected by insufficient specificity of supplementary examinations; moreover, the clinical manifestations may overlap or mislead to different clinical entities in the early stages. Among the subtypes of PSP, the vast majority are associated with two main subtypes—PSP–Richardson's syndrome (PSP–RS) and PSP‐Parkinsonism Predominant (PSP‐P) (Alster et al., 2020b).

Hepcidin is a peptide produced in the liver, primarily defined as a regulator of iron homeostasis, as it regulates its distribution in the tissues (Nicolas et al., 2002). It is linked with disseminated expression throughout the nervous system, including cortical neurons, brain microvascular endothelial cells, and glial cells (Wang et al., 2022). Hepcidin is an element of the nonspecific immune system that plays a role in responding to pathogens. This links hepcidin with the issue of neuroinflammation in the pathogenesis of neurodegenerative diseases (Alster et al., 2020b). Within the evolution of the data concerning the significance of this factor, it was found that it may also impact the patomechanisms of neurodegenerative disorders as Alzheimer's disease (AD) and Parkinson's disease (Sachan et al., 2023). Less is known in the context of its potential significance in atypical parkinsonisms.

## 2 MATERIALS AND METHODS

The study involved the assessment of three groups of participants. The first group consisted of 12 individuals diagnosed with PSP‐RS, comprising 7 males and 5 females, aged between 64 and 75. The second group included 12 patients with PSP‐P, with 7 males and 5 females, aged between 55 and 80. The distinction between the entities was based on the criteria of diagnosis of PSP based on the clinical manifestation (oculomotor dysfunction, akinesia, postural instability, and cognitive/language impairment) (Höglinger et al., 2017). The third group consisted of 12 healthy volunteers, 5 females and 7 males, aged between 35 and 69. The diagnosis of PSP in all cases followed the most recent diagnostic criteria and was conducted by neurologists experienced in movement disorders (Höglinger et al., 2017). The duration of PSP in the participants varied between 3 and 6 years. On average, those with PSP‐P had been dealing with the disease for 3, 5 years, whereas those with PSP‐RS had an average disease duration of 4, 5 years. The study excluded individuals with a history of cancer, infectious diseases, previous strokes, or autoimmune disorders, as specified by the authors (Table 1). All PSP patients underwent examinations at the Department of Neurology of the Medical University of Warsaw using Unified Parkinson's Disease Rating Scale—III part (UPDRS‐III) while in their “OFF” stage. Healthy volunteers underwent examinations at the Department of Infectious and Tropical Diseases and Hepatology Medical University of Warsaw. All of the analyses were done in the Department of Biochemistry of the Medical University of Warsaw. The healthy volunteers were confirmed to be free of any infections or neurological deficits.

**TABLE 1:** Nonspecific parameters among included patients.

	Healthy controls	PSP‐RS	PSP‐P
Ferritin	**Not evaluated**	**No abnormalities detected** 95.8–159.1 ng/mL	**No abnormalities detected** 84.8–125.2 ng/mL
C‐reactive protein	**No abnormalities detected** 0–5 mg/L	**No abnormalities detected** 0–3.1 mg/L	**No abnormalities detected** 0–4.9 mg/L
White blood cells	**No abnormalities detected** 4.10–9.50 × 10^3/uL	**No abnormalities detected** 3.51–8.99 × 10^3/uL	**No abnormalities detected** 3.99–8.07 × 10^3/uL

Abbreviations: PSP–RS, progressive supranuclear palsy–Richardson's syndrome; PSP‐P, PSP‐Parkinsonism Predominant.

The hepcidin level in serum of all patients and controls was measured using the human hepcidin (Hepc) commercial ELISA kit from Cloud‐Clon Corp, according to the manufacturer's instruction. The absorbance was determined using a spectrophotometer at 450 nm (Microplate Spectrophotometer Thermo Scientific Multiskan GO). The levels of hepcidin were calculated based on the standard curve. Both standards and samples were analyzed in duplicate.

Regarding the absence of neuropathological verification of diagnosis, the total levels of tau were additionally evaluated in the CSF. The levels of tau in PSP‐P and PSP‐RS were significantly higher when compared to healthy volunteers. The levels of tau in the CSF did not significantly differ in the comparison of PSP‐P and PSP‐RS.

The analysis results were subjected to statistical evaluation through the utilization of the GraphPad Prisma 8 software. When comparing two tested groups, the Mann–Whitney test was employed. In instances involving more than two groups, quantitative variables were compared using the Kruskal–Wallis test, followed by Dunn's post hoc test for further analysis. Correlations were illustrated using Pearson's coefficient. The presentation of results followed the format of mean ± SD, and statistical significance was established at a threshold of *p* < .05.

## 3 RESULTS

**FIGURE 1:**
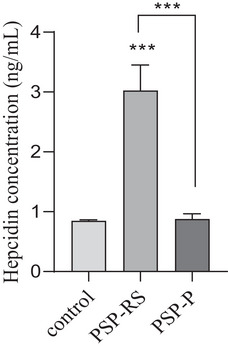
Comparison of serum hepcidin (ng/mL) between patients with progressive supranuclear palsy (PSP)‐RS, PSP‐Parkinsonism Predominant (PSP‐P), and healthy control. Data is expressed as the mean ± SD performed in duplicates. Statistical significance was calculated using the Mann–Whitney *U*‐test. *** *p *< .0001.

**FIGURE 2:**
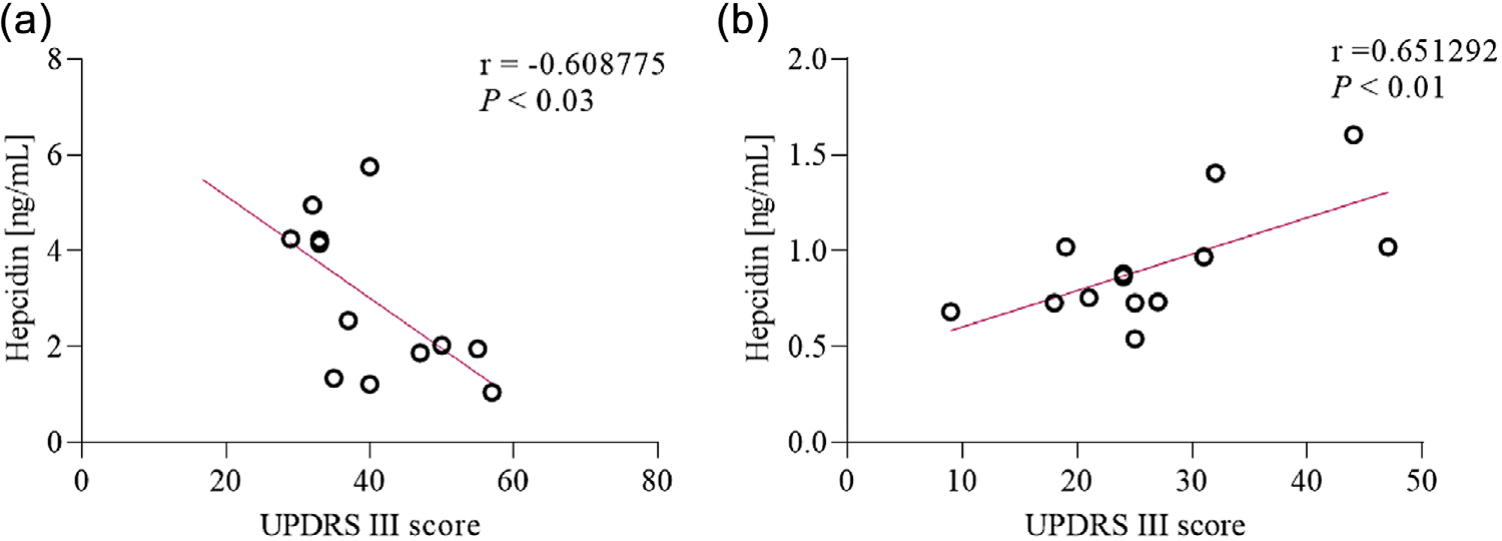
Association of serum hepcidin with Unified Parkinson's Disease Rating Scale—III part (UPDRS‐III) test. Serum hepcidin levels correlated with level of UPRDS III test in progressive supranuclear palsy (PSP)‐RS (a), PSP‐Parkinsonism Predominant (PSP‐P) (b) as determined using Person correlation coefficient (*r_p_*).

Serum hepcidin concentrations were significantly higher (*p* < .0001) only among the PSP‐RS patients (3.10 ± 1.0 ng/mL) as compared to control group (0.84 ± 0.1 ng/mL). No significant difference was observed between PSP‐P in comparison to control group (Figure 1). Significant association was also observed in the subgroup analysis using the Kruskal–Wallis test between PSP‐RS, PSP‐P, and control groups (*p* < .0001). Additionally, post hoc study revealed that hepcidin of level serum was significantly higher in the PSP‐RS group when compared with the control group and PSP‐P (*p* < .0001). Further analysis of serum hepcidin levels revealed correlation with UPDRS‐III results. Serum hepcidin levels were negatively correlated in the PSP‐RS group (*r_p_*  = −0.60, *p* <  =  .023) but in PSP‐P group were positively correlated (*r_p_* = 0.65, *p* < .01) (Figures 2a,b). The assessment of tau was performed only as an element implemented as an element of verification due to the lack of possibility of neuropathological examination as patients are alive.

## 4 DISCUSSION

The results revealed that the possible impact of hepcidin differs depending on the subtype of PSP in two major phenotypes of the disease. Hepcidin is a factor continuously examined in the context of its role in parkinsonisms (Lee & Hyun, 2023; Li et al., 2020; Xu et al., 2022). The links between the distribution of this peptide, dyshomeostasis of iron, links with initiation of neurodegeneration, and dopaminergic toxicity are considered an issue playing a vital role in pathogenesis of this group of diseases (Riederer et al., 2021). The beneficial role of hepcidin in the context of parkinsonian syndrome observed only in PSP‐RS may suggest an alternative mechanism of the disease.

The work shows a significant increase of hepcidin in the serum only in the disease in which the factor may have a beneficial role. In the other subtype—PSP‐P, the level of hepcidin does not significantly differ when compared to controls, which makes the interpretation of the correlation more difficult. On the other hand, hepcidin is more elevated in a more rapidly evolving subtype of the disease with lower life expectancy after diagnosis. This may suggest that the impact of hepcidin in PSP is ambiguous or may differ depending on the feature of the disease, as in this work, authors evaluated only potential correlation between its level and severity of motor deficits. This may partly come up with previous works generally based on evaluation of other neurodegenerative diseases in the context of hepcidin and indicating possible acceleration of neurodegeneration as an effect of metal homeostasis disruption (Myhre et al., 2013; Thomsen et al., 2015). In AD, it was found that hepcidin is associated with lowering of cooper within the cortex (Myhre et al., 2013).

The differences related to the patomechanisms leading to subtypes of PSP are not recognized. PSP‐P is a more favorable clinical entity in the PSP spectrum; however certain works point out that it may be a type of a slower clinical deterioration leading to the same outcome as PSP‐RS. Glial cell‐line‐derived neurotrophic factor, interleukin‐1 and Interleukin‐6 were indicated as factors possibly impacting the evolution of PSP; however, it remains unknown whether the modulation of these elements is a mechanism that may oppose the primary neurodegeneration (Alster et al., 2024; Madetko‐Alster et al., 2023). Other works evaluating possible overlaps between neurodegeneration and inflammatory mechanisms in the pathogenesis of PSP did not indicate the two major subtypes or are based on nonspecific factors as neutrophil‐to‐lymphocyte ratio (Brodacki et al., 2008; Inci et al., 2020; Nübling et al., 2017; Rydbirk et al., 2019).

The work is affected by certain limitations among which could be mentioned lack of neuropathological verification due to the fact that the patients are alive. Authors performed an additional evaluation of the levels of tau in the CSF, which revealed similar levels in PSP‐RS and PSP‐P, which significantly increased when compared to healthy volunteers. The number of the examined patients is relatively low; however, it is associated with the rarity of the disease and the fact that additionally, the examination revealed its subtypes. The work is based on a single evaluation of the level of hepcidin and examination using UPDRS‐III in “OFF” stage. Moreover, the clinical evaluation was performed using a scale not dedicated to the examination of PSP due to the fact that the PSP rating scale was not implemented in polish. The age of the control group was significantly lower; however, the level of hepcidin was not found to be correlated with age.

## 5 CONCLUSION

The role of hepcidin in PSP is not specified. As hepcidin is a factor negatively correlated with UPDRS‐III results in the context of deficit, it may be partly considered a factor linked with preservation of motor functions of patients with PSP‐RS. Hepcidin should not be interpreted as a fully protective factor as other features of the disease were not evaluated. In PSP‐P, the observation suggesting the increase of hepcidin as a feature linked with the more pronounced severity of motor symptoms may be affected by significant limitations, among which could be mentioned the fact that the level of this peptide in PSP‐P does not significantly differ when compared to volunteers. This outcome, combined with the limited number of patients, should encourage further research in the field based on larger groups and based on evaluation of various aspects of the disease. The results of the work are a preliminary analysis of the potential impact of hepcidin in certain forms of atypical parkinsonism.

## AUTHOR CONTRIBUTIONS

**Piotr Alster**: Conceptualization; data curation; formal analysis; funding acquisition; investigation; methodology; project administration; resources; supervision; validation; writing‐original draft; writing—review and draft. **Dagmara Otto‐Ślusarczyk**: Formal analysis; software; writing‐original draft; writing—review and draft. **Alicja Wiercińska‐Drapało and Marta Struga**: Project administration; supervision; validation; writing‐original draft; writing—review and draft. **Natalia Madetko‐Alster**: Data curation; formal analysis; funding acquisition; investigation; methodology; project administration; resources; supervision; validation; writing‐original draft; writing—review and draft.

## CONFLICT OF INTEREST STATEMENT

The authors declare that there are no conflicts of interest relevant to this work.

### PEER REVIEW

The peer review history for this paper is available at https://publons.com/publon/10.1002/brb3.3552.

## Data Availability

The data is available from the corresponding author on request.
